# Self-report occupational-related contact dermatitis: prevalence and risk factors among healthcare workers in Gondar town, Northwest Ethiopia, 2018—a cross-sectional study

**DOI:** 10.1186/s12199-019-0765-0

**Published:** 2019-02-14

**Authors:** Tesfaye Hambisa Mekonnen, Dawit Getachew Yenealem, Beyene Mindaye Tolosa

**Affiliations:** Department of Environmental and Occupational Health and Safety, Institute of Public Health, College of Medicine and Health Sciences, University of Gondar, P.O. Box 196, Gondar, Ethiopia

**Keywords:** Occupational-related contact dermatitis, Healthcare workers, Self-report, Ethiopia

## Abstract

**Background:**

Occupational skin diseases are the second most common occupational diseases and are responsible for an estimated 25% of all lost work days. Occupational contact dermatitis (OCD) comprises 70–90% of all occupational skin diseases. In Ethiopia, information about the prevalence and factors which determine developments of contact dermatitis is not recognized. The objective of this study was to investigate prevalence and factors influencing the occurrences of occupational-related contact dermatitis among healthcare workers in Gondar town, Northwest Ethiopia.

**Methods:**

We employed a healthcare-based cross-sectional study from March to April 2018. A stratified sampling method followed by simple random sampling method was used to select 422 participants. The standardized Nordic Occupational Skin Questionnaire was pretested and interviewer-administered for data collection. We used SPSS version 20 to conduct a binary logistic regression analysis. We set ≤ 0.05 *p* value to ascertain significance and 95% CI with odds ratios to evaluate the strength of associations.

**Results:**

Response rate was 100%. The majority, 52.4% (*N* = 221), were males. The mean age was 22.6 (SD ± 6.3) years. The overall prevalence of self-report occupational contact dermatitis in the previous 12 months was 31.5% (*N* = 133) [95% CI (27, 36.2)]. The highest symptoms indicated was redness, 28.5% (*n* = 38), followed by burning, 17.3% (*n* = 23). The hand is the most commonly affected body sites, 22% (*N* = 93). Hand washing frequency [AOR 1.80, 95% CI (1.10, 3.20)], pairs of hand gloves used per day [AOR 3.22, 95% CI (2.05, 5.87)], personal history of allergy [AOR 2.37, 95% CI (1.32, 4.61)], and lack of health and safety training [AOR 2.12, 95% CI (1.12, 2.25)] were factors considerably associated with contact dermatitis.

**Conclusions:**

The prevalence of occupational-induced contact dermatitis is common among healthcare workers in Ethiopia. Therefore, our finding indicates that intervention aiming at workers’ health and safety training demands urgent public health responses to tackle the ailment. The result also demonstrates that healthcare workers should be aware of when and how hands should be washed. The number of pairs of gloves used per day should also be taken into consideration while devising prevention strategies.

## Background

Occupational skin disease is the second most common occupational diseases [1, 2]. According to the Health and Safety Executive (HSE) report, there are around 16,000 cases every year in the UK [3]. In 2010, approximately, there had been 850,000 cases of work-related dermatitis among workers in the USA [4]. Occupational skin diseases are responsible for an estimated 25% of all lost work days [5]. Occupational contact dermatitis (OCD) accounts for 70–90% of all occupational skin diseases, which deteriorates functional capacity and the quality of life [6]. It is an inflammation of the skin caused by exposure to substances in the workplace [3, 7–9]. The most common symptoms include swelling, itching, flaking or cracking of the skin, blisters, and weeping sore of skin [3].

Occupational contact dermatitis is the most common form of work-related skin diseases usually experienced by health professions [10, 11]. Working in healthcare is regarded as a risk factor for occupational-related skin diseases [2, 12–14]. Workers are often exposed to cleaning materials, like disinfectants, soaps, detergents, latex, and thorough and frequent hand washing [11, 12]. The use of alcohol gel, contact with allergens, and the occlusive effect of gloves also lead to contact dermatitis in healthcare professions [6]. A study demonstrated that using latex gloves can predispose to the developments of contact dermatitis among healthcare workers [13].

The prevalence of contact dermatitis is usually seen between 10 and 40%, in general [2]. A study from Greece delineated that 39.9% of the sampled employees suffered from occupational dermatitis [15]. A study conducted in Poland showed that prevalence of skin disorders among healthcare workers ranges from 41 to 86% [13].

In Ethiopia, together with the recent advent of healthcare system developments, employment rate of healthcare workers is rapidly growing but with little/or no protection of their health and safety. Exposure to various healthcare-related hazards that increase the likelihood of experiencing symptoms of occupational-related contact dermatitis is, therefore, usually remarkable. Despite the problem pervasiveness, the magnitude and risk factors influencing work-related contact dermatitis among healthcare workers is often unnoticed. The objective of the current study was, therefore, to investigate the prevalence and risk factors associated with the occurrences of occupational-related contact dermatitis among healthcare workers in Gondar town, Northwest Ethiopia. An investigation of prevalence and risk factors for occupational-induced dermatitis is imperative to understand the etiology of disease and inform better preventive strategies.

## Methods

### Study design and period

We conducted a cross-sectional study from March to April 2018 to assess prevalence and identify the factors affecting work-related contact dermatitis among healthcare workers.

### Study setting and area

This study was conducted among healthcare workers in Gondar town, Northwest Ethiopia. Gondar town is located 748 km to the northwest of Addis Ababa, the capital of Ethiopia. There are two hospitals (a public and private) in the town employing more than 700 healthcare workers. We included the two hospitals purposively to attain the required sample size.

### Source population

All healthcare workers working in the hospitals in Gondar town were our source population.

### Inclusion and exclusion criteria

#### Inclusion criteria

We included all healthcare workers who had been working in the hospitals for at least 12 months prior to the study period.

#### Exclusion criteria

Administrative, supportive, and nonclinical (no direct contact with patients) staffs were excluded.

### Sample size and sampling procedures

We employed a stratified sampling technique to select the participants. A single population proportion was used to calculate the required sample size. A 50% assumption for prevalence and an absolute precision of 5% were considered. We also assumed 95% confidence level to obtain adequate power for analysis. After considering an additional 10% for nonresponse rates, 422 participants were included in the study.

### Operational definitions

Work-related contact dermatitis: A noninfectious disease caused by skin contact (either allergic or irritant contact) with substances used at work, with any of the symptoms of contact dermatitis, including redness, burning, blisters, itching, dry skin, fissures, aching or pain, and crusting that appeared in any part of the body in the previous 12 months [16]

Healthcare workers (HCWs): Included health officers, nurses, midwives, medical laboratory technologists, medical doctors, pharmacists, psychiatrists, and optometrists who work in clinical departments (have direct contact with patients) in the hospitals

Body mass index (BMI): Workers weight (w) divided by height squared (h^2^) where:

< 18.5: underweight

18.5–24.99: normal

≥ 25: overweight/obesity

A satisfied worker with a job: A generic job satisfaction scale score of 32 or above [17]

A stressed worker with a job: A workplace stress scale score of 21 or above [18]

### Data collection tools and techniques

We collected data using a structured interviewer-administered questionnaire. A self-report contact dermatitis was assessed by the standardized Nordic Occupational Skin Questionnaire version 2002 (NOSQ-2002) [19]. Perceived job satisfaction was assessed by a generic job satisfaction scale questionnaire [17]. We assessed perceived job stress using a job stress scale questionnaire [18]. We divided the components of the questionnaire into four parts. The first part covered socio-demographic characteristics, like sex, age, educational status, profession, marital status, monthly salary, and work experience. The second part covers work-related factors, including working hours per day, department, preemployment and periodic medical examination, types of glove used, pairs of gloves used per day, frequencies of hand washing, shift work, health and safety training, overtime (working more than 8 h per day), utilization of hand gloves in days per week, and utilization of hand gloves in hours per day. The third part of the questionnaire constitutes the detailed information about self-report history of chronic diseases, such as atopic fever (yes/no), hay fever (yes/no), asthma (yes/no), childhood dermatitis (yes/no), personal and family history of allergy (yes/no), and rhinitis (yes/no). The last category of the survey questionnaire covered behavioral factors, like physical exercise (yes/no), smoking (yes/no), and body mass index (BMI) (weight divided by height squared).

### Data quality control

To ensure the quality of data collected, we gave much emphasis to the appropriate design of data collection tools. First, the questionnaire was designed in English and translated into the local language “Amharic” and back to English by language experts. Second, we recruited three data collectors and two supervisors who had previous experience and skills in the task. We trained and oriented them for 2 days before the actual data survey. The training content included about the clarity of questionnaire and purposes of the study, the confidentiality of information, informed consent, and the roles and responsibilities of the data collectors as well as supervisors. The principal investigator supervised the overall data collection tasks. Third, we conducted a pretest on 10% of the sample in a neighboring hospital, Kola Diba, prior to the actual data collection days to test the validity and consistency of the instrument used. We modified some words and misinterpretations, minimized the number of questions, and made corrections to some other objections.

### Data management and analysis

Completeness of data was checked on a regular basis during the data collection process. We coded data, labeled, verified, categorized, and entered into EpiInfo version 7 software. We used SPSS version 20 to analyze data and computed frequencies, percentages, means, and the standard deviation to present findings. The reliability of data collection instrument was checked and found that the reliability of the instrument was acceptable with Cronbach’s alpha score of 0.988. A bivariate logistic regression analysis was performed separately for each independent variable to explore the associations with the dependent variable (occupational contact dermatitis). The explanatory variables which were significant at < 0.2 *p* values in a bivariate analysis were exported to the multivariable logistic regression model to control the potential effects of confounders. Variables were dropped into the multivariate logistic regression model with a forward variable selection method. We checked the goodness of fit model using Hosmer and Lemeshow and found the assumption satisfied (*p* value > 0.05). A cut off ≤ 0.05 *p* value was set to evaluate the significance and odds ratios (OR) with 95% confidence interval (CI) to establish the strength of associations.

## Results

### Socio-demographic characteristics

A total of 422 healthcare professionals participated with a response rate of 100%. The majority of the respondents, 52.4% (*N* = 221), were males. The mean age was 22.6 (SD ± 6.3) years. In a high proportion, 74.9% (*N* = 316) were married and 39.8% (*N* = 168) were nurses (Table 1).

**Table 1:** Socio-demographic characteristics of HCWs in Gondar town, Ethiopia, 2018

Variables (*N* = 422)	Frequency	Percent (%)
Sex		
Male	221	52.4
Female	201	47.6
Age		
18–24	14	3.3
25–35	192	45.5
≥ 36	216	51.2
Religion		
Orthodox	236	55.9
Muslim	125	29.6
Protestant	49	11.6
Others+	12	2.8
Marital status		
Single	92	21.8
Married	316	74.9
Separated/divorced/widowed	14	3.3
Educational level		
First degree (BSc, MD)	174	41.2
Masters (not medical) and specialists	248	58.8
Monthly salary in BIRR		
≤ 4000	140	33.2
4001–4999	148	35
≥ 5000	134	31.8
Profession		
Nurse	168	39.8
Midwives	138	32.7
Laboratory technologists	64	15.2
Dentists	3	.7
Surgeon	16	3.8
Others*	33	7.8
Work experience		
< 5 years	194	46
5–10 years	104	24.6
> 10 years	124	29.4

*Others+* Catholic, Juba; Others* pediatricians, anesthesia, optometrists, psychiatrists; pharmacists; health officers; medical doctors; *HCWs* healthcare workers; *BSc* Bachelor of Science; *MD* medical doctor; *BIRR* Ethiopian currency

### Work-related characteristics

**Fig. 1:**
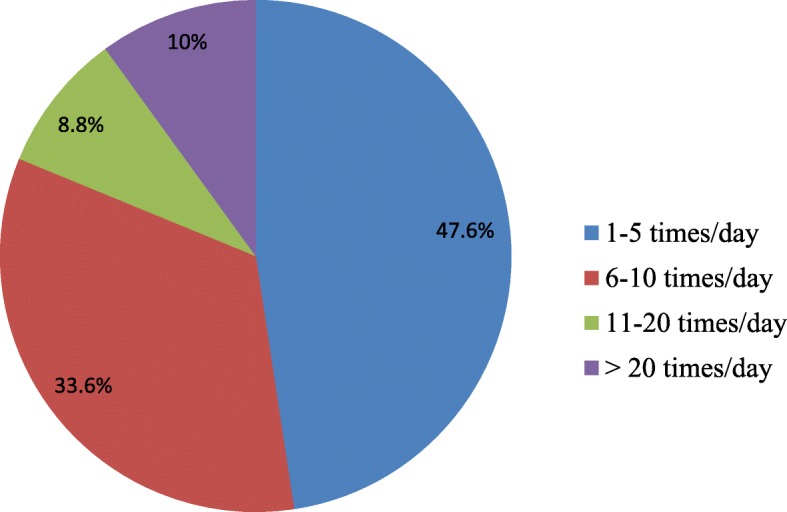
Hand washing frequencies among healthcare workers in Gondar town, Ethiopia, 2018 (*N* = 422)

Out of the participants, 78.9% (*N* = 333) indicated that they had been working for ≤ 8 h per day. Less than half of the respondents, 49.8% (*n* = 210), described that they worked overtime (more than 8 h per day). Thirty-five percent (*N* = 150) of the participants showed that they had worked shift work (night and day shifts). The majority, 71.8% (*N* = 303), said that they had not received any training on health and safety issues. Almost half of the study sample, 50.9% (*N* = 215), demonstrated that there was no periodic medical examination services in their workplace. The majority of the participants, 98.8% (*N* = 417), reported that they used some types of personal protective equipment (PPE) for their activities. Almost all, 99.3% (*N* = 419), of the workers had illustrated that they always used gloves for their jobs. Regarding the type of gloves used, 58.8% (*N* = 248) described that they used natural rubber/latex glove, whereas 37.2% (*N* = 157) and 4% (*N* = 17) indicated that they used synthetic rubber and plastic types of gloves, respectively. Among the respondents, 38.6% (*N* = 163) reported that they used gloves for < 2 h per day. Forty-one percent (*N* = 173) said that they used gloves for 2–6 h per day, whereas 20.4% (*N* = 86) for > 6 h per day. Of the participants, 47.6% (*N* = 201) reported that they wash their hands one to five times per day (Fig. 1).

### Prevalence of self-report occupational contact dermatitis

**Fig. 2:**
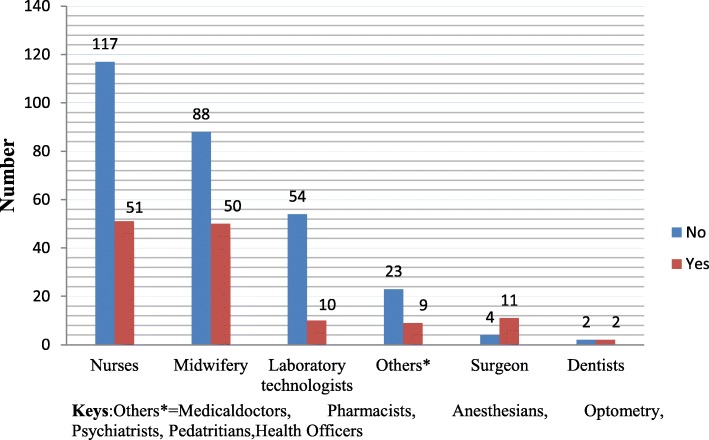
Distribution of occupational contact dermatitis by profession, Gondar town, Ethiopia, 2018 (*N* = 422)

**Fig. 3:**
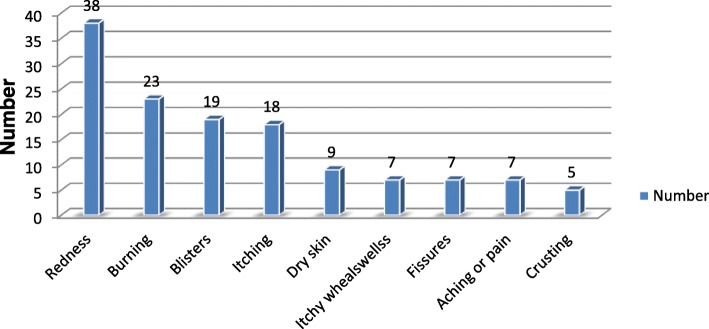
Perceived symptoms of contact dermatitis among healthcare workers in Gondar town, Ethiopia, 2018 (*n* = 133)

The overall prevalence of self-report work-related contact dermatitis in the previous 12 months was 31.5% (*N* = 133) [95% CI (27, 36.2)]. Nurses indicated a high proportion of contact dermatitis, 12.1% (*N* = 51), followed by midwifery professionals, 11.8% (*N* = 50) (Fig. 2)**.** Redness was showed to be the highest symptoms of self-report contact dermatitis, 28.6% (*n* = 38), followed by burning, 17.3% (*n* = 23) (Fig. 3). A high proportion of the symptom of self-report dermatitis was observed on the hands (hand dermatitis), 22.0% (*N* = 93). Five percent (*N* = 19) of the participants reported having had experienced contact dermatitis on their faces. The prevalence of contact dermatitis among healthcare workers was 2.1% (*N* = 9) and 2.8% (*N* = 12) on the eyes and other body parts, respectively. Of the victims, 17.3% (*n* = 23) indicated that they had experienced work-related contact dermatitis in more than one body sites.

### Occupational characteristics of the problem

Of the reported contact dermatitis, 75.9% (*n* = 101) indicated that their symptoms last for more than 3 weeks. Regarding the occupational relatedness of the problem, 92.5% (*n* = 123) said that their symptoms become made worse when they contact with certain materials, chemicals, and anything else at their workplaces. Twenty-six percent (*n* = 34) of the participants who reported contact dermatitis indicated that contact with certain materials outside their work aggravated their symptoms. Most of the participants who indicated the problem, 96.2% (*n* = 128) said that their symptoms improve on days away from work.

### Factors affecting the occurrences of contact dermatitis

A bivariate logistic regression analysis showed that education, monthly salary, work experience, working hours per day, frequency of hand washing per day, job satisfaction, pairs of hand gloves used per day, periodic employment medical examination, hours of hand gloves used per day, health and safety training, and having personal previous history of allergy were significantly associated with occupational contact dermatitis.

In the multivariable logistic regression analysis, however, the frequency of hand washing per day, pairs of hand glove used per day, health and safety training, and previous history of allergy remained to significantly affect the occurrences of contact dermatitis. Our finding demonstrated that hand washing frequency significantly affected the development of contact dermatitis. The participants who washed their hands 11 and more times per day were 1.80 times more likely to develop contact dermatitis than those who washed 5 and fewer times a day [AOR 1.801, 95% CI (1.10, 3.20)]. The pairs of hand glove used per day indicated to significantly affect contact dermatitis. The odds of having contact dermatitis were 3.22 times high among respondents who used five and more pairs of gloves per day than those who used a pair of gloves per day [AOR 3.22, 95% CI (2.05, 5.87)]. Having a previous history of allergy also importantly influenced the development of contact dermatitis. Respondents who indicated to have been diagnosed with allergy in the previous time were 2.37 times more likely to develop occupational-related contact dermatitis than those who did not indicate as having a previous history of allergy [AOR 2.37, 95% CI (1.32, 4.61)]. Having health and safety training was the other factors which importantly affected the experiences of occupational contact dermatitis. The participants who received no training about workplace health and safety were 2.12 times more likely to develop contact dermatitis than those who received training on health and safety [AOR 2.18, 95% CI (1.12, 2.25)] (Table 2).

**Table 2:** Factors affecting occupational contact dermatitis (OCD) among HCWs, Gondar town, 2018, Ethiopia

Variables (*N* = 422)	Contact dermatitis		COR (95% CI)	AOR (95% CI)	*p* value
	Yes	No			
Educational level					
First degree (BSc, MD)	39	135	2.11 (1.6, 3.28)	1.41 (0.84, 2.30)	0.06^+^
Masters (not medical) and specialists	94	154	1	1	
Monthly salary in BIRR					
≤ 4000	30	110	2.79 (1.65, 4.75)	1.11 (0.03, 2.10)	0.041^+^
4001–4999	45	103	1.60 (0.94, 2.73)	1.01 (0.08, 1.49)	
≥ 5000	58	76	1	1	
Work experience					
< 5 years	45	149	1	1	0.079^+^
5–10 years	31	73	1.41 (0.82, 2.40)	1.40 (0.02, 2.19)	
> 10 years	57	67	2.82 (1.73, 4.58)	1.40 (0.04, 1.59)	
Working hours per day					
≤ 8	73	260	1	1	0.057^+^
> 8	60	29	7.37 (4.41, 12.32)	3.80 (0.11, 4.82)	
Hand washing frequency per day					
≤ 5 times	35	150	1	1	0.001*
6–10 times	29	109	1.14 (0.66, 1.98)	1.11 (0.85, 1.22)	
> 10 times	69	30	9.86 (5.60, 17.34)	1.80 (1.102, 3.20)	
Job satisfaction					
Satisfied	73	278	1	1	0.061^+^
Not satisfied	60	11	20.77 (10.39, 41.52)	5.36 (0.18, 8.19)	
Pairs of hand gloves used per day					
< 1 pair	15	104	1	1	0.002*
1–5 pairs	43	172	1.73(.92, 3.28)	1.53 (0.43, 3.12)	
> 5 pairs	75	13	40.0 (17.98, 89.01)	3.22 (2.05, 5.87)	
Periodic medical examination					
Yes	78	137	1	1	0.048^+^
No	55	152	1.56 (1.03, 2.37)	1.23 (0.87, 1.09)	
Personal history of allergy					
Yes	46	10	14.75 (7.15, 30.46)	2.37 (1.32, 4.61)	0.001*
No	87	279	1	1	
OSH training					
No	114	209	2.31 (1.59, 3.89)	2.12 (1.12, 2.25)	0.001*
Yes	19	80	1	1	
Hours of hand gloves used per day					
< 2 h/day	47	116	1	1	
2–6 h/day	51	122	1.03 (0.31, 2.11)	1.01 (0.15, 1.93)	0.079^+^
> 6 h/day	35	51	1.70 (0.18, 3.25)	1.51 (0.39, 2.65)	0.053^+^

1 represents a reference group. All variables we presented in this table were included in the multivariable model

*AOR* adjusted odds ratios, *CI* confidence interval, *COR* crude odds ratio, *HCWs* healthcare workers, *BSc* Bachelor of Science, *MD* medical doctor, *N* number, *OSH* occupational safety and health

^+^Significant in a bivariate analysis

*Significant in a multivariable analysis

## Discussion

Occupational contact dermatitis is a priority occupational-related health problem markedly affecting employees’ quality of life and performance efficiency. This study employed a healthcare-based cross-sectional study to investigate the prevalence and risk factors influencing the occurrences of work-related contact dermatitis among healthcare workers in Gondar town, Northwest Ethiopia. The prevalence of self-report occupational-related contact dermatitis in the previous 12 months was 31.5% (*N* = 133) [95% CI (27.0, 36.2)]. This result was comparable with a study in Namibia (31.3%) [20]. This might be due to similarities in socioeconomic characteristics and poor implementation of workplace health and safety services in these regions, as developing countries. We found a lower prevalence of occupational-related contact dermatitis than a study report in Lithuania (47.3%) [21], Turkey (61.7%) [22], Bulgaria (58.5%) [23], and Greece (39.9%) [15]. The differences might be due to the differences in workplace illness management and reporting cultures and procedures available between the countries.

Our finding was, however, higher compared to a study finding in China (28.5%) [24], Bulgaria (28.2%) [25], and Taiwan (8%) [26]. This may be due to disparities in the characteristics of study participants and setting among the countries. Hand dermatitis (hand eczema), 22% (*N* = 93), was the most commonly indicated occupational-induced contact dermatitis in the current study. This magnitude was lower than that of previous reports in Japan [27] and India [28] (36.2% prevalence in both) and higher than that of a study in Saudi Arabia (7.73%) [29]. The difference may be due to differences in sample size and method of data collection employed. Redness, 28.5% (*n* = 38), was the most common symptoms of all occupational-related contact dermatitis observed in our investigation. This was lower than literature report in the UK (49%) [30]. The difference could be due to the differences in illness reporting cultures and data collection methods used.

This study revealed that having previous history of personal allergy significantly influenced the likely occurrences of occupational-related contact dermatitis. This finding was in line with other reports [5, 22, 24, 27, 29]. The possible explanation might be due to that having a personal previous history of allergy might exacerbate the occurrence of contact dermatitis. A more plausible reason is that having a previous history of allergy may lead to the developments of contact dermatitis, particularly allergic contact dermatitis due to stimulation of the body’s mechanisms, for example, IL-4/Th2 pathway [31, 32], increasing the individual susceptibility to elicitation response to environmental triggers.

In the current study, frequency of hand washing per shift considerably affected the experience of occupational contact dermatitis. This result was equivalent to other study findings [6, 15, 24, 27]. This may be due to that repeated hand washing may expose workers to an extraordinary amount of contact with water (wet work) and soap/cleansing agents that can affect the normal outer layer of skin. Repetitive hand washing exposes to constant wetting and drying, removing protective substances from the skin that makes it less pliable and more prone to contact dermatitis related to occupation.

Our study also indicated that pair of hand gloves used per day was an important risk factor for work-related contact dermatitis. This finding agreed with other studies [13, 21, 33]. A possible explanation might be that the barrier and exposure protection function of the skin may be impaired by occlusion effects of wearing pairs of gloves.

Lack of health and safety training was the other significant factor for occupational-related contact dermatitis. There is scant research that indicates the association between occupational-related contact dermatitis and workers’ training status on workplace health and safety issues. A study conducted in India corroborated with this finding [28]. The possible suggestion for our finding is due to the fact that health and safety training plays an important preventive role from different occupational related ill health, including occupational-related contact dermatitis. It is often evident that workplace health and safety training likely increase workers’ knowledge and awareness towards prevention and control of the risks and hazards associated with adverse health conditions, as this generally boosts early recognition and notification of the conditions. Moreover, employees’ provision with safety training at the earliest possible period of employment might also improve workplace safety cultures and practices. Previous reports also noted that occupational health and safety education encourages employees to recognize and report health conditions at the earliest possible [34, 35]. In Ethiopia, coverage in health and safety service is empirically very poor. Health and safety training is thus rarely implemented, urging to the likely developments of health conditions, including that of work exposure-related occupational contact dermatitis. The authors would like to recommend future researchers to further examine the associations between the lack of health and safety training and occupational-related contact dermatitis.

This study would likely contribute considerable evidence to literature regarding prevalence and the factors influencing occurrences of work-related contact dermatitis. However, few limitations have not been ruled out. First, the study was based on a cross-sectional design. Therefore, it might be difficult to conclude the temporal relationship between the outcome of interest (occupational-related contact dermatitis) and factors influencing its occurrences. Next, the study was based on respondents’ self-report data. As a result, underestimation of the condition due to recall bias may be expected. Moreover, the finding was not supported by clinical diagnoses, like patch testing that help to identify work-related irritant and allergic contact dermatitis. However, we used the standardized Nordic Occupational Skin Questionnaire that was validated for estimating a self-report prevalence of skin dermatitis in a given population. Last, while translating the data collection tool from English to the local language (Amharic), some technical errors may occur. But to minimize such errors, the translation was done by language experts.

## Conclusions

Occupational-related contact dermatitis is the common work-related skin problem among healthcare workers in Ethiopia. Hand washing frequency, pair of hand gloves used per day, having personal history of allergy, and the lack of health and safety training were suggested to be the significant risk factors. Therefore, our findings indicate that there is a greater need for healthcare workers for better safety and health training at the workplaces. Awareness creation for healthcare workers on when and how hands should be washed is highly recommended. The number of pairs of gloves used per day should also be taken into consideration to tackle the ailment.

## Abbreviations

AOR: Adjusted odds ratios
BMI: Body mass index
BSc: Bachelor of Science
CD: Contact dermatitis
CI: Confidence interval
COR: Crudes odds ratio
HCWs: Healthcare workers
HSE: Health and Safety Executive
MPH: Masters of Public Health
NOSQ: Nordic Occupational Skin Questionnaire
OR: Odds ratios
SPSS: Statistical Package for Social Sciences
UK: United Kingdom
